# Dual diagnosis clients' treatment satisfaction - a systematic review

**DOI:** 10.1186/1471-244X-11-64

**Published:** 2011-04-18

**Authors:** Sabrina J Schulte, Petra S Meier, John Stirling

**Affiliations:** 1 International Studies Department, American University of Sharjah, P.O. Box: 26666, Sharjah, United Arab Emirates; 2 School of Health and Related Research, University of Sheffield, 30 Regent Street, Sheffield, UK; 3 Department of Psychology, Elizabeth Gaskell Campus, Manchester Metropolitan University, Manchester, UK

**Keywords:** Dual diagnosis, co-morbidity, integrated treatment, mental illness, satisfaction, substance misuse

## Abstract

**Background:**

The aim of this systematic review is to synthesize existing evidence about treatment satisfaction among clients with substance misuse and mental health co-morbidity (dual diagnoses, DD).

**Methods:**

We examined satisfaction with treatment received, variations in satisfaction levels by type of treatment intervention and by diagnosis (i.e. DD clients vs. single diagnosis clients), and the influence of factors other than treatment type on satisfaction. Peer-reviewed studies published in English since 1970 were identified by searching electronic databases using pre-defined search strings.

**Results:**

Across the 27 studies that met inclusion criteria, high average satisfaction scores were found. In most studies, integrated DD treatment yielded greater client satisfaction than standard treatment without explicit DD focus. In standard treatment without DD focus, DD clients tended to be less satisfied than single diagnosis clients. Whilst the evidence base on client and treatment variables related to satisfaction is small, it suggested client demographics and symptom severity to be unrelated to treatment satisfaction. However, satisfaction tended to be linked to other treatment process and outcome variables. Findings are limited in that many studies had very small sample sizes, did not use validated satisfaction instruments and may not have controlled for potential confounders. A framework for further research in this important area is discussed.

**Conclusions:**

High satisfaction levels with current treatment provision, especially among those in integrated treatment, should enhance therapeutic optimism among practitioners dealing with DD clients.

## Background

The evidence base regarding best practice for the treatment of clients with co-occurrence of substance misuse and mental health problems (dual diagnosis, DD) remains ambiguous. While some studies have found promising client outcomes after integrated treatment (simultaneous care for both problem areas by the same provider) [1-3], several systematic reviews have concluded that the evidence remains inconsistent as to whether integrated care is more effective than parallel or sequential treatment approaches [4-10].

While most DD studies evaluate treatment effectiveness in terms of improvements in clinical outcomes (i.e. severity of substance misuse and/or psychiatric symptoms), recent research has also started to focus on client perceptions of treatment. Clients' views towards their care have been commonly subsumed under the term of 'treatment satisfaction', which refers to "the extent to which a programme is perceived as having met an individual's treatment wants and needs" (p. 456) [11]. Examining client satisfaction can provide valuable insights into treatment delivery by identifying the nature and extent of unmet needs and expectations [12-15]. Client perceptions are increasingly recognised as an important indicator of treatment quality with previous research showing links between satisfaction, treatment adherence, retention and clinical treatment outcomes [16-23]. Recent treatment guidelines in both the mental health and the addiction field list the improvement of client satisfaction as a key target [24-26]. Taking into account the ongoing uncertainty around best-practice models for the DD population, clients' own treatment perceptions about different care approaches may be important in identifying potential problems in the quality of existing interventions and in informing future treatment developments.

### Objectives

The aim of this review is to synthesize existing evidence about treatment satisfaction among DD clients. The following four key questions have guided the review:

1) How satisfied are DD clients with treatment they receive?

2) Do satisfaction levels among DD clients differ according to whether they receive integrated or standard care?

3) Do DD clients report lower treatment satisfaction levels compared to single diagnosis clients when treated in the same clinical setting?

4) Do studies identify other factors related to treatment satisfaction in DD clients?

## Methods

### Study inclusion criteria

**Figure 1:**
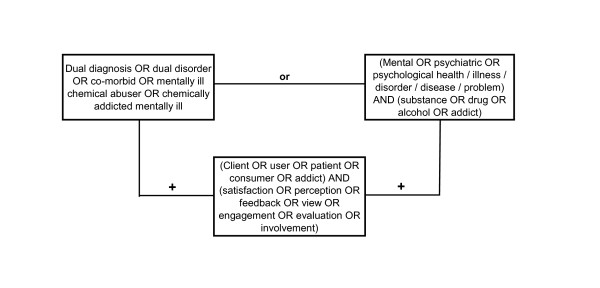
**Search terms used for electronic databases and other sources**.

We considered all quantitative studies that assessed treatment satisfaction among adult clients with co-existing drug/alcohol misuse and mental health problems, without placing restrictions on the clinical setting in which the study was carried out or the type of diagnosis procedure used. Studies were excluded if sample sizes were smaller than N = 10, treatment provision comprised self-help groups only or was limited to a single treatment session. Furthermore, studies had to provide basic information about the satisfaction assessment used and to report results of DD clients' satisfaction ratings separately from any other groups that may also have been investigated. The electronic databases PsycInfo, Medline, Academic Search Premier and ProQuest were searched using pre-defined search strings to identify studies published in English-language peer-reviewed journals between January 1970 and October 2010 (see Figure 1). Bibliographies and citation records of relevant papers were also examined.

### Selection of potentially relevant studies

**Figure 2:**
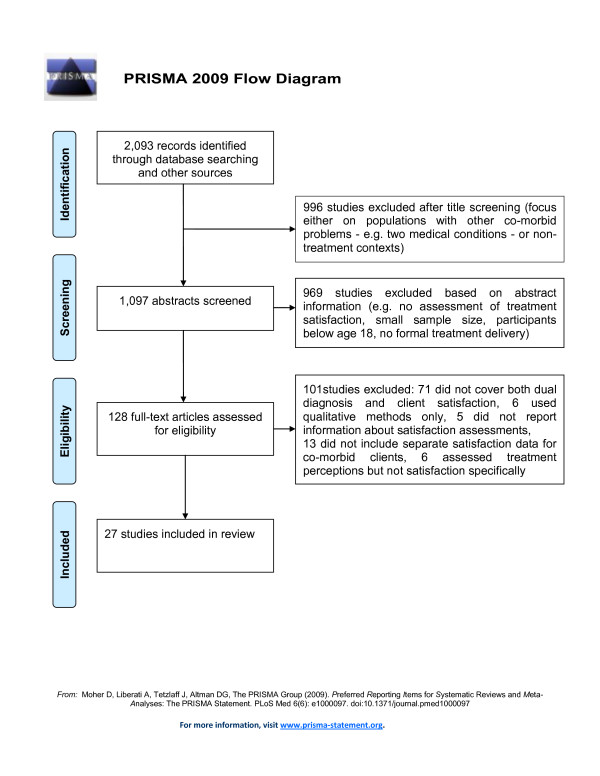
**Study selection process**.

Initially, search results (N = 2,093) were screened based on study titles by the first author. Studies were excluded if titles indicated that the focus was on populations with other co-morbidities (e.g. two medical conditions) or non-treatment contexts (n = 996, see Figure 2). Next, abstracts of the remaining studies were examined (n = 1,097) to decide if a study met the inclusion criteria and appeared to address at least one of the research questions. As a result, 969 studies were excluded based on the information given in the abstract (e.g. small sample size, participants younger than 18 years). The full text article was obtained for 128 studies, which were subjected to a more detailed analysis using a self-developed data extraction form (available from the first author). That is, relevant information (e.g. methods used for assessing satisfaction, sample size, research questions addressed) was extracted from each of the 128 articles to determine their eligibility for the current review. In order to avoid missing relevant studies, full texts were also screened for DD-related articles where it was unclear from the abstract whether treatment satisfaction was assessed (e.g. range of outcome variables not fully specified). At this stage, 101 studies were excluded because they i) did not explicitly focus on both co-morbidity and client satisfaction together (n = 71), ii) did not separately report satisfaction levels among client subgroups with DD problems (n = 13), iii) used qualitative methods only (n = 6), iv) assessed client perceptions but not treatment satisfaction explicitly (n = 6), and v) did not provide sufficient detail about the satisfaction instrument used (n = 5). Full citation details for these studies are given in Additional File 1.

We assessed the quality of each of the remaining 27 studies selected for inclusion by critically appraising the following aspects of its protocol based on existing guidelines [27]: study design (e.g. single vs. multiple satisfaction assessment points), research instruments used (standardised vs. non-standardised), adequacy of a study's sample size (e.g. power calculations mentioned) and robustness of analytic approach (e.g. control variables). Furthermore, we intended to include a meta-analysis of those studies that address research question 2 and 3. However, due to the small number of studies available, difficulties in the data preparation process (i.e. statistics required for calculating effect measures were missing in two articles), and high heterogeneity among studies, we considered a quantitative synthesis of data inappropriate for the current review.

## Results

### Description of included studies

Of the 27 included studies, 21 were conducted in the US, four in the UK, one in Australia and one in Honduras. All studies reported treatment satisfaction ratings of DD clients (research question 1) and seven studies compared such ratings by type of treatment intervention provided (research question 2). Only three studies could be found that investigated whether or not satisfaction ratings differ among clients with or without DD problems when treated in the same setting (research question 3). Nine studies reported testing for links between additional factors (e.g. client demographics) and treatment satisfaction in DD clients (research question 4). Of the 27 studies, two represented updates or extensions of earlier studies conducted in the UK [28,29] and the US [30,31]. In these cases, it was not possible to establish the extent to which subject pools overlapped, so both updated study reports were included in the current review.

Sample sizes ranged from 17 to 2,729 clients (see Additional File 2). Most studies included clients with co-morbidity only. Five studies compared satisfaction levels between DD clients and those with either mental health or substance misuse problems only [32-36]. Types of treatment setting and interventions delivered varied greatly across studies, ranging from residential psychiatric or addiction services to forensic programmes and assertive integrated treatment models (see Additional File 2).

#### Client profiles

The majority of participants were male. Four studies included men only [36-39] and four others examined women only [40-43]. The mean ages ranged from 30 to 45 years (SD = 6.3-14.0). Six US studies appeared to recruit disproportionately more African-Americans than Caucasians [30-32,37,44,45] whilst in eight other studies Caucasian was the most common ethnic group [35,40,46-51]. In the remaining studies, client ethnicity was diverse. The sample studied by Aguilera et al. [38] differed in ethnicity from all others due to its location (Honduras). Five studies included homeless individuals only [30,31,37,48,51] and two focused on clients involved in the criminal justice system [39,47]. Information about clients' socio-demographics was incomplete for a number of study reports [33,39,45,46,52,53].

Turning to the type of mental illnesses identified, 12 of the 27 studies mainly included clients who suffered from schizophrenia-spectrum disorders or other severe mental illnesses with psychotic features [28-32,34,37,39,45,46,49,52]. Four studies investigated participants with posttraumatic stress disorder or histories of abuse in addition to mental health problems [40-43] and another focused on personality disorders [47]. The ten remaining studies reported affective or anxiety disorders as the most common mental illnesses [33,35,36,38,44,48,50,51,53,54].

In terms of substance use, 14 studies identified alcohol as being the main substance of misuse [28-31,33,36,40,47-52,54]. In two other studies cocaine was the most common drug [44,45], in one study methamphetamine [42] and in another cannabis [39]. Two studies described most participants as polydrug users [37,38]. Seven studies did not include details about clients' primary substance [32,34,35,41,43,46,53].

#### Assessment of treatment satisfaction

Of the 27 studies identified, 13 reported the use of a standardised instrument for assessing treatment satisfaction (see Table 1 and Additional File 2). All but one of these employed the Client Satisfaction Questionnaire (CSQ-8) [55] either in its original form or with minor modifications. In six of these 13 studies, additional assessment instruments were adopted (e.g. Treatment Perceptions Questionnaire) [11]. The remaining 14 studies did not employ standardised satisfaction measures and used either single items asking clients about their satisfaction with the overall treatment experience (n = 4) or multiple items covering several aspects related to treatment delivery (n = 10).

**Table 1:** Clients' mean scores of treatment satisfaction ratings including standard deviation

Author	Instrument (score range)	Mean score	Standard Deviation
Afuwape et al. (2006) [28]	CSQ-8 (8-32) TPQ (0-40)	CSQ-8: 21.5-21.5, TPQ: 19.9-23.8	CSQ: 5.3-6.9; TPQ: 5.2-7.2

Aguilera et al. (1999) [38]	Unknown measure (not applicable^1^)	9.6	Not reported

Anderson (1999) [37]	Unknown measure (not applicable^1^)	Not reported; 85-88% somewhat to very satisfied	Not reported

Boden & Moos (2009) [36]	Modified CSQ (0-33)	25.2-26.7	5.4-6.3

Brown et al. (2007) [43]	Self-developed scale (-3 to +3)	2.3-2.7	0.7-0.9

Burns et al. (2005) [33]	Self-developed scale (1-4)	2.7-2.8	Not reported

Clark et al. (2008) [41]	CSQ-8 (8-32); CPC (26-104)	CPC: 76.7; CSQ: not reported	CPC: 12.4; CSQ: not reported

Covington et al. (2008) [42]	CSQ-8 (8-32)	Not reported; 92% positive to very positive ratings	Not reported

Craig et al. (2008) [52]	CSQ-8 (8-32); TPQ (0-40)	CSQ-8: 22.8-23.5; TPQ: 20.1-21.5	CSQ: 5.7-6.5; TPQ: 0.8-8.6

Daughters et al. (2008) [44]	Modified CSQ (8-32)	24.6-27.6	2.8

Godley et al. (2000) [47]	Self-developed scale (1-5)	4.2-4.3	0.6

Harrison et al. (2008) [48]	Self-developed scale (not reported)	Not reported; 92% satisfied to very satisfied	Not reported

Herrell et al. (1996) [35]	Self-developed scale (1-7)	4.8-5.1	Not reported

Magura et al. (2008) [53]	Self-developed scale (0-10)	7.5	2.7

McHugo et al. (1999) [49]	Modified Lehman's QOL Interview (1-7)	4.9-5.2	0.9-1.2

Miles et al. (2003) [29]	CSQ-8 (8-32); TPQ (0-40)	CSQ: 21.7-23.7; TPQ: 18.5-22.6	CSQ: 4.8-6.6; TPQ: 6.8-8.9

Miles et al. (2007) [39]	Self-developed scale (not reported)	Not reported; 88-100% satisfied	Not reported

Moore et al. (2009) [50]	Self-developed scale (not reported)	Not reported; 75-90% satisfied to very satisfied	Not reported

Morse et al. (2006) [30]	Self-developed scale (1-6)	4.7-5.2	0.7-1.0

Morse et al. (2008) [31]	Self-developed scale (1-6)	4.2-5.1	0.4-1.1

Najavits et al. (1998) [40]	Modified CSQ (1-4)	3.0-3.1	0.4

Pollack et al. (1997) [54]]	CSQ-8 (8-32)	27.5	0.7-0.9

Primm et al. (2000) [32]	CSQ-8 (8-32)	24.7-28.3	1.9-4.5

Prince (2005) [34]	Self-developed scale (1-4)	Not reported; > 89% satisfied	Not reported

Ries et al. (1999) [46]	Modified CSQ (4-20)	16.6	0.9-1.1

Shaner et al. (2003) [45]	Self-developed scale (1-5)	Not reported; scores of > 4 on all items	Not reported

Wise (2010) [50]	CSQ-8 (8-32)	29.6	Not reported

Key: CSQ-8 = Client Satisfaction Questionnaire (8 items), TPQ = Treatment Perceptions Questionnaire, CPC = Consumer Perceptions of Care, QOL = Quality of Life. For more details about all instruments, see Additional File 2.

^1 ^20 Fill-in-the-blank questions were used.

More than half of the studies (n = 17) assessed clients' treatment satisfaction at a single point in time only (see Additional File 2). Of these, ten studies provided information about the length of treatment stay when client satisfaction was measured. The other ten studies included in this review obtained satisfaction data repeatedly and at different treatment stages, ranging from baseline to 36-month follow-up assessments.

### How satisfied are DD clients with currently available treatment options?

Clients consistently reported high average satisfaction scores, which in some studies were close to the maximum score of the scales used, thus suggesting that on the whole, clients tend to be satisfied with their treatment (see Table 1 and Additional File 2). Direct comparisons of satisfaction scores between study samples are problematic due to the diversity of assessment instruments used, differences in client profiles, treatment settings, interventions delivered and study designs. While variability of satisfaction scores was low in most studies, greater differences in ratings between clients were found in the three UK-based studies, which additionally used the Treatment Perceptions Questionnaire [28,29,52] (see Table 1). In another study where greater variability in the overall mean satisfaction score was also shown, the scale that was used covered several aspects beyond treatment satisfaction, which complicates the interpretation of the score range [41] (see Table 1 and Additional File 2). Turning to the stability of client ratings over time, those studies that assessed satisfaction at multiple treatment stages (n = 10, see Additional File 2) reported no significant changes in ratings across assessment points. For one of these studies [30] though, updates from a 30-month follow-up assessment were published in which a trend of decreasing satisfaction levels over time was reported [56]. It remained unclear if this decline reached an acceptable level of statistical significance as no probabilities were reported.

### Do satisfaction levels among DD clients differ by type of treatment model?

All seven studies that investigated associations between type of treatment approach and satisfaction ratings compared a form of integrated DD treatment (i.e. simultaneous care for both the mental health and substance misuse problems by the same provider) with standard care models without specific DD focus [30,31,37,38,41,44,52]. The range of specific interventions and settings of the integrated treatment programmes differed to some extent across the seven studies (e.g. depression- vs. trauma-focused care and residential vs. assertive settings; see Table 2 and Additional File 2).

**Table 2:** Satisfaction levels among dual diagnosis clients by type of treatment model

Author	Sample	Treatment intervention	Control condition	Satisfaction levels between groups	Treatment fidelity
Aguilera et al. (1999) [38]	N = 86 Main DD: mood disorder + polydrug misuse	DD treatment (n = 40)	Drug/alcohol treatment (n = 46)	No difference in treatment satisfaction scores. Results of statistical tests not reported.	Not reported

Anderson (1999) [37]	N = 225 Main DD: psychosis + polydrug misuse	DD treatment (n = 76)	Drug/alcohol treatment (n = 149)	Higher satisfaction levels among intervention group (n = 42) but relevant tests not reported.	Not reported

Clark et al. (2008) [41]	N = 2,729 Main DD: unspecified + history of trauma	Trauma-focused DD treatment (n = 1,415)	Mental health or drug/alcohol treatment (n = 1,314)	Intervention group had higher satisfaction scores at follow-ups (3-month: F = 8.77, p < 0.01; 6-month: F = 4.07, p < 0.05).	Not reported

Craig et al. (2008) [52]	N = 232 Main DD: psychosis + alcohol misuse	DD treatment (n = 127)	Mental health treatment (n = 105)	No significant differences in satisfaction levels (CSQ: p = 0.39, TPQ: p = 0.62).	Not reported

Daughters et al. (2008) [44]	N = 44 Main DD: mood and anxiety disorders + cocaine misuse	Depression-focused DD treatment (n = 22)	Drug/alcohol treatment (n = 22)	The intervention group reported significantly higher satisfaction levels (p < 0.01).	High levels of treatment fidelity (mean = 7.3 on 9-point Likert scale).

Morse et al. (2006) [30]	N = 149 Main DD: schizophrenia + alcohol misuse	Assertive DD treatment (IACT; n = 46)	1. Assertive mental health treatment (ACTO; n = 54) 2. Standard mental health or drug / alcohol treatment (SC; n = 49)	Clients in the IACT and ACTO programme were significantly more satisfied than SC clients (p = 0.03). ^1, 2^	Treatment diffusion between IACT and ACTO. ^3^

Morse et al. (2008) - based on [30] - [31]	N = 270 Main DD: schizophrenia + alcohol misuse	New assertive DD treatment (NIACT; n = 79)	1. IACT (n = 61) 2. ACTO (n = 65) 3. SC (n = 65)	Clients in the NIACT programme were significantly more satisfied than clients in the other 3 programmes (p < 0.001).	High level of treatment fidelity in the NIACT model. ^4^

Key: DD = dual diagnosis

^1 ^No significant differences in satisfaction levels between the IACT and ACTO groups (no statistics reported). No main effect of time (p = 0.32).

^2 ^Updated findings of this study were published by Fletcher et al. (2008) including results from additional satisfaction assessments: 3 months: IACT = 5.10 (0.72), ACTO = 5.23 (0.84), SC = 4.76 (1.06), 15 months: IACT = 4.79 (1.18), ACTO = 5.10 (1.16), SC = 5.00 (0.95), and 30 months: IACT = 4.20 (0.35), ACTO = 4.15 (0.52), SC = 4.36 (0.38).

^3 ^Treatment fidelity of different service components was measured using 5-point Likert scales. Treatment diffusion between IACT and ACTO: substance abuse components were only partially implemented in IACT, evidence of addiction-focused interventions and DD training in ACTO.

^4 ^Mean fidelity scores ranged from 3.9-4.1 using 5-point Likert scales (same as in Morse et al. 2006).

The earliest of these seven studies compared satisfaction ratings of 42 male clients treated in a residential integrated programme with 93 clients receiving residential addiction treatment only [37]. Participants provided satisfaction ratings one month after treatment discharge. Information about clients' average length of treatment stay was not reported. Results showed that the majority (88%) of clients in the integrated treatment programme were satisfied with their care (46% = 'very satisfied', 42% = 'somewhat satisfied'). Similar overall satisfaction rates were found in the comparison group (85%), but here less than one quarter (23%) said they were 'very satisfied' and almost two thirds (62%) reported being 'somewhat satisfied' (see Additional File 2). The lack of a standardised satisfaction measure means that the results cannot easily be compared with other studies. Despite reporting that the differences found were statistically significant, the author did not include relevant test results.

The same author was part of a research team that replicated the above-mentioned study in Honduras [38]. Here, 40 male DD clients based in residential integrated treatment were compared with 46 clients treated in a residential drug/alcohol programme on a range of outcome variables including satisfaction. The satisfaction assessment took place three months after treatment intake or upon successful completion and discharge from the programme. The authors reported identical average satisfaction scores for both treatment groups (see Table 1 and Additional File 2).

In a large multi-site study [41] 1,415 female clients were provided with integrated trauma-focused treatment, compared to 1,314 participants who received standard care. At the 3- and 6-month follow-up assessments, the intervention group had significantly higher satisfaction ratings than the controls (see Table 2 and Additional File 2). The study used a newly developed measure to assess client views. This instrument had high internal consistency (α> 0.9) and was moderately correlated with the CSQ-8 (r = 0.56, p < 0.001, n = 121). However, clients' satisfaction scores on the CSQ-8 were not reported separately.

Similar findings were shown by a smaller recent study [44], which compared 22 DD clients who received two weeks of integrated inpatient care with a DD control group (n = 22) provided with standard drug/alcohol treatment. All participants were randomly allocated to the two treatment interventions and satisfaction was assessed after a treatment stay of five weeks. The intervention group reported significantly higher satisfaction levels (see Table 2). In sum, all but one of the above-mentioned studies showed that DD clients receiving integrated care were more satisfied than the comparison groups in standard treatment.

The other three studies that compared satisfaction levels between DD clients who were provided with either integrated or standard care were conducted in outpatient treatment settings. The most recent study took place in the UK and had the advantage of using two different satisfaction measures (see Additional File 2) [52]. The authors examined whether clients (n = 45) treated in an integrated fashion by practitioners with DD training were more satisfied than clients (n = 86) provided with community mental health treatment by non-trained practitioners. No differences in satisfaction levels between the two client samples were found at the18-month follow-up assessment (see Table 2).

In contrast, a US study [30] showed that 46 DD clients treated by staff who had received training in delivering assertive integrated treatment were significantly more satisfied than 49 clients treated in general non-assertive addiction or mental health programmes. This difference was evident throughout four assessment points between six and 24 months after treatment initiation and was maintained at the recently reported 30-month assessment [56]. Nevertheless, satisfaction ratings were very similar in the assertive DD-focused treatment condition and a third comparison group (n = 54) of assertive mental health-focused treatment (see Table 2 and Additional File 2). Hence, study results might suggest that participants who received assertive treatment had higher satisfaction levels than participants in a non-assertive treatment programme regardless of whether or not there was a DD focus. At the same time however, the authors noted that some treatment overlap occurred between the DD and mental health-focused assertive treatment conditions during the study period. That is, the two programmes were less distinct than intended (i.e. substance abuse components were only partially implemented in the integrated treatment group and there was evidence of addiction-focused interventions and DD training in the mental health programme). Therefore, it is possible that the lack of differences in satisfaction ratings between those two programmes is due to the actual treatment provided being quite similar.

This assumption is supported by findings from a study that built upon and extended the above-mentioned approach [31]. Here, a new assertive integrated treatment condition was added, in which 79 clients were provided with extra addiction-focused services aiming to achieve higher treatment fidelity. Clients in this fourth treatment group reported significantly greater satisfaction at three and 15 months after intake than clients in the other two assertive treatment programmes and the control condition (p < 0.001, see Table 2 and Additional File 2).

The assessment of treatment fidelity by measuring the extent to which interventions were implemented as intended is a particular strength of the two studies conducted by Morse and colleagues [30,31]. Apart from the previously mentioned study by Daughters et al. [44], where therapists' adherence to the treatment manual was monitored and confirmed to be high, none of the other studies that compared satisfaction in DD clients by treatment type reported data on treatment fidelity (see Table 2). Another strength of these three studies is that clients were randomly allocated to the different treatment conditions. In contrast, client selection biases due to non-randomization have to be considered in the other four studies described in this section.

### Are DD clients less satisfied compared to non-DD clients when treated in the same clinical setting?

Three studies were identified that addressed this question (see Table 3 and Additional File 2). In a recent large US study [36], treatment satisfaction with a residential drug/alcohol programme was measured in male clients with and without co-morbid problems (n = 691 and n = 1,805, respectively). The authors reported that DD clients were significantly less satisfied with treatment than the comparison group at discharge.

**Table 3:** Satisfaction levels among DD and non-DD clients in same treatment setting

Author	Treatment setting	Total sample	DD clients	Non-DD clients	Satisfaction levels between groups
Boden & Moos (2009) [36]	Drug/alcohol programme	N = 2,496	n = 691 Main DD: mood disorder + alcohol misuse	n = 1,805 Problem area: alcohol misuse	DD clients were significantly less satisfied with treatment (F = 27.9, p < 0.01).

Burns et al. (2005) [33]	Drug/alcohol programme	N = 71	n = 48 Main DD: mood disorder + alcohol misuse	n = 23 Problem area: alcohol misuse	No significant differences in satisfaction scores between groups (t = -0.41, p = 0.15).

Herrell et al. (1996) [35]	Mental health programme	N = 92	n = 24 Main DD: mood disorder + unspecified substance misuse	n = 68 Problem area: mood disorder	No significant differences in satisfaction scores between groups (t = 1.14, p > 0.25).

Key: DD = dual diagnosis

In contrast, two earlier smaller-scale studies had shown no significant differences: In a US study, severely mentally ill clients in a residential mental health facility were classified either as having DD problems (n = 24) or suffering from mental illness only (n = 68) [35]. Several measurements were taken pre- and post-treatment (approximate treatment length was three weeks) including client satisfaction after discharge. The non-DD sample had a slightly higher mean satisfaction score than the DD group, but the difference was not statistically significant (see Table 3 and Additional File 2). Similarly, an Australian study carried out in two drug/alcohol outpatient programmes asked 71 participants to provide satisfaction ratings three months after treatment intake [33]. Again, results showed no statistically significant differences in satisfaction scores between DD (n = 48) and non-DD clients (n = 23; see Table 3 and Additional File 2). No power calculations were reported and these two studies may have been too small to detect moderate group differences.

### Other factors linked to treatment satisfaction among DD clients

Several studies reported investigating associations between satisfaction, client and treatment-related factors in their DD samples. The selection of test variables (e.g. clients' gender, frequency of service contacts) differed across studies hence complicating direct comparisons. Studies that examined client socio-demographics found no link between gender, age, education, employment, marital status or ethnicity and treatment satisfaction [28,34,41,54]. Similarly, there were no associations between primary substance used or type of psychopathology and satisfaction [29,34].

A number of studies examined associations between satisfaction and other treatment-related variables. One study found a weak but significant positive relationship between greater satisfaction levels, clients' own outcome ratings (r = 0.3, p < 0.05) and case managers' evaluations of clients' progress (r = 0.3, p < 0.05) [46]. Another study reported that provision of staff assistance to clients' family members in coping with the individuals' mental illness significantly increased treatment satisfaction (OR = 6.91, p < 0.05) [34]. No programme-specific analyses were mentioned by the authors (i.e. satisfaction ratings in programmes with family assistance vs. programmes without family assistance) but an effect was apparent at the client level (i.e. all clients who received such assistance from any of the programmes were more satisfied). More recently it was shown that satisfaction was associated with clients' ratings of the treatment's usefulness for their recovery (r = 0.6, p < 0.05) [53]. Furthermore, this study found both satisfaction and treatment usefulness ratings to be correlated with another variable referred to as 'Changes in Recovery Behaviours' (e.g. reduced substance use, taking psychiatric medications, self-care; multiple R = 0.3, p < 0.05). Findings from these studies need to be considered carefully though, as it remained unclear whether or not other potentially confounding variables (e.g. client motivation, therapeutic alliance) were included in the analyses.

Nevertheless, the results above are partially supported by a well-controlled study [56] which found that treatment satisfaction was positively influenced - though to a varying extent over time - by the intensity of help with activities of daily living, help with emotional problems and transportation assistance. Further variables associated with satisfaction were the frequency of contact with the programme in general and the number of service contacts where substance misuse issues were addressed specifically [56]. All mentioned variables were linked to higher treatment satisfaction across the three treatment programmes included in the study (i.e. after controlling for treatment condition).

Moving from treatment process to outcome variables, one study demonstrated the positive effect of client satisfaction on clinical outcomes, including reduced substance misuse problems and psychiatric symptom severity at both 1- and 5-year follow-ups, after controlling for a range of potential confounders [36].

## Discussion

Over the last four decades, 27 studies meeting our inclusion criteria could be identified that examined treatment satisfaction in DD clients. This review shows that most DD clients report being satisfied with their treatment experience, reflected by average ratings close to the "satisfied" end of the scales used. This applied regardless of the differences in study location (i.e. US, UK, Australia or Honduras), treatment settings and types of interventions delivered. When comparing satisfaction ratings of dual and single diagnosis clients treated in the same setting (i.e. either mental health or substance misuse treatment), a large and well-designed study found that DD clients were significantly less satisfied than single diagnosis clients [36]. Two smaller studies, however, showed that clients with co-morbid problems had similarly high satisfaction ratings as those with a single diagnosis [33,35]. This inconsistency may be linked to differences in satisfaction instruments used (i.e. standardised vs. non-standardised), client profiles (e.g. the larger study included men only) and the small sample sizes in the two studies that found no differences in satisfaction ratings (N < 50).

If replicated in future studies, a finding that DD clients are less satisfied with standard (i.e. either mental health or addiction-focused) treatment than single diagnosis clients would support the common understanding that disease-specific treatment is inadequate to address the complex needs of the DD population. An integrated treatment model is usually favoured in discussions about which approach is the most beneficial for co-morbid clients e.g. [57,58]. The question as to whether or not these benefits are also reflected in greater satisfaction levels was specifically addressed by seven studies included in this review. Of these, five offered evidence that integrated care yields greater client satisfaction than standard treatment [30,31,37,41,44]. The five studies were all conducted in the US whereas the two other studies that found no significant differences in satisfaction by treatment approach were carried out elsewhere (UK and Honduras ) [38,52]. In this context, however, it is important to bear in mind that of the seven studies identified only three assessed treatment fidelity and thus monitored if the integrated treatment condition was implemented as intended. These three studies consistently demonstrated higher satisfaction levels in the integrated treatment group compared to ratings from clients in standard care [30,31,44].

Nine studies investigated which factors - other than treatment type - are associated with satisfaction among DD clients. Studies that examined client pre-treatment factors (i.e. demographics, primary substance of misuse and type of psychopathology) found no association with satisfaction ratings. In contrast, a number of treatment process and service-related variables were identified that appeared linked to satisfaction (e.g. client and staff outcome ratings, frequency of contact with treatment service, family and transportation assistance). In some studies though, it remained unclear whether or not potential confounders were taken into account, which needs to be addressed in future studies. Moreover, it would be important to examine the effect of variables that have been found to be associated with treatment satisfaction among single diagnosis samples in the past (e.g. access routes, treatment motivation and engagement, care-plan procedures, staff and service characteristics) [11,59-65].

In terms of rigor, the 27 studies were diverse, and some had important methodological shortcomings. Only 13 studies used standardised measures to assess treatment satisfaction, and while the selected instruments have shown acceptable psychometric properties when used with single diagnosis treatment populations e.g. [11,55,66,67], the scales' reliability and validity in clients with co-morbidity was reported by only two studies [36,41]. DD clients might have different treatment expectations due to more complex needs than those with a single diagnosis. Thus, response patterns to a given set of questions might vary between populations with and without DD, and psychometric testing would be important to ensure meaningful interpretation of data. Similarly, only three of the studies that used a self-developed satisfaction scale provided psychometric information sufficient to permit reasonable evaluation of the instruments [30,31,51].

Secondly, studies were restricted in their examination of potential confounders of satisfaction ratings. Only five studies reported explicitly that they controlled for any links between client characteristics and satisfaction levels [28,34,36,41,54]. The lack of client control variables and other potential confounders (e.g. treatment process variables, practitioner characteristics) is of particular concern in those studies that compared satisfaction levels by type of treatment model: uncontrolled factors may affect clients' satisfaction ratings, which in turn distorts interpretations concerning actual treatment effects.

A third methodological difficulty concerns possible time-in-treatment effects on satisfaction ratings. In most of the reviewed studies, clients were at different treatment stages when satisfaction was assessed, with only ten studies taking the length of treatment exposure into account. Two of these reported client satisfaction at different treatment stages, with one showing stable high ratings throughout [40] and the other study indicating a negative linear trend in satisfaction levels during the treatment course [56]. Based on the latter, it could be assumed that clients' most urgent needs are addressed in the early treatment phase thus producing particularly high satisfaction levels early on in the programme. In later treatment phases though, possibly more persistent problem areas are targeted for which behaviour change and improvement is more difficult to achieve. Subsequently, studies examining satisfaction early in treatment may find higher satisfaction ratings than studies with later assessment schedules. However, at the same time it is plausible that clients who have spent more time in treatment may have experienced greater benefits overall and possibly show higher satisfaction levels than clients who have spent less time in the programme [68]. In either case, having more information about potential time-in-treatment effects across the existing studies would have been useful.

The current review has highlighted some important gaps in our knowledge of treatment satisfaction among DD clients such as the influence of practitioner characteristics and treatment process variables as well as the effect of client satisfaction on different treatment outcomes. Clients' subjective evaluations have been recognised in both mental health and addiction treatment populations as key indicators of treatment quality and effectiveness e.g. [19,21,36,69], and so this remains an important area of research. The review contributes a methodological framework of four key aspects that future studies should consider to overcome the limitations, namely: 1) employment of well-validated and comparable satisfaction assessment techniques, 2) selection of multiple measures that incorporate several treatment- and client-related factors, 3) controlling for potential confounders of satisfaction, including pre- and in-treatment factors (e.g. treatment readiness, frequency of service contact, substitute prescribing) and practitioner characteristics (e.g. work experience), and 4) the nature and extent of treatment exposure (e.g. assertive vs. standard care, length of treatment stay). Here, special attention should be paid to the assessment of treatment fidelity. This is particularly important for studies aiming to replicate the finding that integrated treatment - if implemented appropriately - yields greater client satisfaction than other treatment models. Furthermore, it would be vital for future studies to investigate links between satisfaction and other treatment process and outcome variables to demonstrate more clearly whether greater satisfaction among DD clients translates into better engagement and retention, lower relapse rates and reduced symptom severity. Finally, a more general point requires consideration: a recent review has shown that satisfaction studies disproportionally found positive accounts from clients throughout treatment modalities and client populations [70]. In order to avoid misinterpretation of client ratings due to social desirability or other potential bias, safeguards should be applied in future studies, such as keeping assessments anonymous and comparing satisfaction ratings of treatment completers and dropouts.

A limitation of the current review is that no meta-analysis could be carried out. A quantitative synthesis of data could have taken into account small sample sizes and moderate - if not significant - effects thus providing further insight into the current evidence base. Depending on the growth of studies in this field, future reviews should include such analyses where possible.

## Conclusions

Our review shows that dually diagnosed clients are, on the whole, satisfied with current treatment provision, despite the common notion that individuals with co-morbidity are the most difficult-to-treat clients e.g. [71]. Integrated treatment delivery, which simultaneously addresses both addiction and mental health concerns, appeared to result in particularly high levels of satisfaction. Findings should be of particular interest to treatment providers as it may enhance optimism among practitioners dealing with such clients.

## Competing interests

The authors declare that they have no competing interests.

## Authors' contributions

SJS carried out the literature search, examined all records obtained, interpreted the data and drafted the manuscript. PSM assisted in the literature search and made substantial contributions to the evaluation of selected articles and manuscript draft. JS was involved in revising the draft in several stages. All authors read and approved the final manuscript.

## Pre-publication history

The pre-publication history for this paper can be accessed here:

http://www.biomedcentral.com/1471-244X/11/64/prepub

## Supplementary Material

Additional File 1 **Studies on dual diagnosis clients and treatment satisfaction that were excluded after full-text retrieval**. Shows full citations for those studies that did not meet the review's eligibility criteria Click here for file

Additional File 2 **Overview of studies assessing treatment satisfaction among dually diagnosed clients**. Shows key characteristics of all studies included in the review Click here for file
